# Rationale, design and baseline characteristics of the effect of ticagrelor on health outcomes in diabetes mellitus patients Intervention study

**DOI:** 10.1002/clc.23164

**Published:** 2019-04-09

**Authors:** Deepak L. Bhatt, Kim Fox, Robert A. Harrington, Lawrence A. Leiter, Shamir R. Mehta, Tabassome Simon, Marielle Andersson, Anders Himmelmann, Wilhelm Ridderstråle, Claes Held, Philippe Gabriel Steg, on behalf of the THEMIS Steering Committee, R. Diaz, J. Amerena, K. Huber, P. Sinnaeve, J.C. Nicolau, J.F.K. Saraiva, I. Petrov, L.A. Leiter, S.R. Mehta, R. Corbalán, J. Ge, Q. Zhao, R. Botero, P. Widimský, S.D. Kristensen, J. Hartikainen, N. Danchin, H. Darius, T.H. Fat, R.G. Kiss, P. Pais, E. Lev, L.D. Luca, S. Goto, G.A.R. López, J.H. Cornel, F. Kontny, F. Medina, N. Babilonia, G. Opolski, D. Vinereanu, D. Zateyshchikov, M. Ruda, O. Elamin, F. KováŘ, A.J. Dalby, M.H. Jeong, H. Bueno, S. James, C.‐E. Chiang, D. Tresukosol, Z. Ongen, K. Ray, A. Parkhomenko, D. McGuire, M. Kosiborod, T.Q. Nguyen

**Affiliations:** ^1^ Brigham and Women's Hospital Heart and Vascular Center Harvard Medical School Boston Boston Massachusetts; ^2^ National Heart and Lung Institute Imperial College and Royal Brompton Hospital London UK; ^3^ Stanford Center for Clinical Research (SCCR), Department of Medicine, Stanford University Stanford California; ^4^ Li Ka Shing Knowledge Institute, St. Michael's Hospital University of Toronto Toronto Ontario Canada; ^5^ Population Health Research Institute Hamilton Health Sciences and McMaster University Hamilton Ontario Canada; ^6^ AP‐HP, Hôpital Saint Antoine, Department of Clinical Pharmacology‐URCEST Sorbonne‐Université Paris Paris France; ^7^ AstraZeneca Gothenburg, Department of Cardiovascular, Renal and Metabolism Mölndal Sweden; ^8^ Department of Medical Sciences, Cardiology, Uppsala Clinical Research Center Uppsala University Uppsala Sweden; ^9^ FACT (French Alliance for Cardiovascular Trials), an F‐CRIN Network, Département Hospitalo‐Universitaire FIRE, AP‐HP, Hôpital Bichat Université Paris‐Diderot Paris France; ^10^ Département Hospitalo‐Universitaire FIRE, AP‐HP, Hôpital Bichat,Université Paris‐Diderot, INSERM U‐1148 Paris France; ^11^ National Heart & Lung Institute NHLI, Imperial College, Royal Brompton Hospital London UK; ^12^ Argentina; ^13^ Australia; ^14^ Austria; ^15^ Belgium; ^16^ Brazil; ^17^ Brazil; ^18^ Bulgaria; ^19^ Canada; ^20^ Canada; ^21^ Chile; ^22^ China; ^23^ China; ^24^ Colombia; ^25^ Czech Republic; ^26^ Denmark; ^27^ Finland; ^28^ France; ^29^ Germany; ^30^ Hong Kong; ^31^ Hungary; ^32^ India; ^33^ Israel; ^34^ Italy; ^35^ Japan; ^36^ Mexico; ^37^ Netherlands; ^38^ Norway; ^39^ Peru; ^40^ Philippines; ^41^ Poland; ^42^ Romania; ^43^ Russia; ^44^ Russia; ^45^ Saudi Arabia; ^46^ Slovakia; ^47^ South Africa; ^48^ South Korea; ^49^ Spain; ^50^ Sweden; ^51^ Taiwan; ^52^ Thailand; ^53^ Turkey; ^54^ UK; ^55^ Ukraine; ^56^ USA; ^57^ USA; ^58^ Vietnam

**Keywords:** antiplatelet therapy, clinical trials, diabetes mellitus, general clinical cardiology/adult, ischemic heart disease

## Abstract

In the setting of prior myocardial infarction, the oral antiplatelet ticagrelor added to aspirin reduced the risk of recurrent ischemic events, especially, in those with diabetes mellitus. Patients with stable coronary disease and diabetes are also at elevated risk and might benefit from dual antiplatelet therapy. The Effect of Ticagrelor on Health Outcomes in diabEtes Mellitus patients Intervention Study (THEMIS, NCT01991795) is a Phase 3b randomized, double‐blinded, placebo‐controlled trial of ticagrelor vs placebo, on top of low dose aspirin. Patients ≥50 years with type 2 diabetes receiving anti‐diabetic medications for at least 6 months with stable coronary artery disease as determined by a history of previous percutaneous coronary intervention, bypass grafting, or angiographic stenosis of ≥50% of at least one coronary artery were enrolled. Patients with known prior myocardial infarction (MI) or stroke were excluded. The primary efficacy endpoint is a composite of cardiovascular death, myocardial infarction, or stroke. The primary safety endpoint is Thrombolysis in Myocardial Infarction major bleeding. A total of 19 220 patients worldwide have been randomized and at least 1385 adjudicated primary efficacy endpoint events are expected to be available for analysis, with an expected average follow‐up of 40 months (maximum 58 months). Most of the exposure is on a 60 mg twice daily dose, as the dose was lowered from 90 mg twice daily partway into the study. The results may revise the boundaries of efficacy for dual antiplatelet therapy and whether it has a role outside acute coronary syndromes, prior myocardial infarction, or percutaneous coronary intervention.

## 1 INTRODUCTION

Type 2 diabetes mellitus (DM) is a highly prevalent risk factor for coronary artery disease, with an incidence that is increasing worldwide. In those who have established atherosclerosis, the presence of DM further increases future risk of ischemic events in a synergistic fashion.^1^ DM multiplies cardiovascular (CV) risk not only in those with prior ischemic events, but also in those with stable coronary artery disease.

Heightened platelet activity appears to be present in atherothrombotic patients with DM. Long‐term dual antiplatelet therapy (DAPT) reduces CV event rates in patients with acute coronary syndromes, both in the short‐ and long term.^2^, ^3^, ^4^, ^5^, ^6^, ^7^, ^8^, ^9^, ^10^, ^11^ Dual antiplatelet therapy appears to have a particular benefit in patients with prior myocardial infarction (MI) and diabetes.^12^, ^13^, ^14^, ^15^ Whether that benefit extends to patients with diabetes and stable coronary artery disease without a history of prior MI remains a major unanswered question.

The Effect of Ticagrelor on Health Outcomes in diabEtes Mellitus patients Intervention Study (THEMIS) trial was designed to evaluate the potential benefits and risks of dual antiplatelet therapy with ticagrelor plus low dose aspirin vs placebo plus aspirin in patients with established stable coronary artery disease and DM treated with medications.

## 2 METHODS

**Figure 1:**
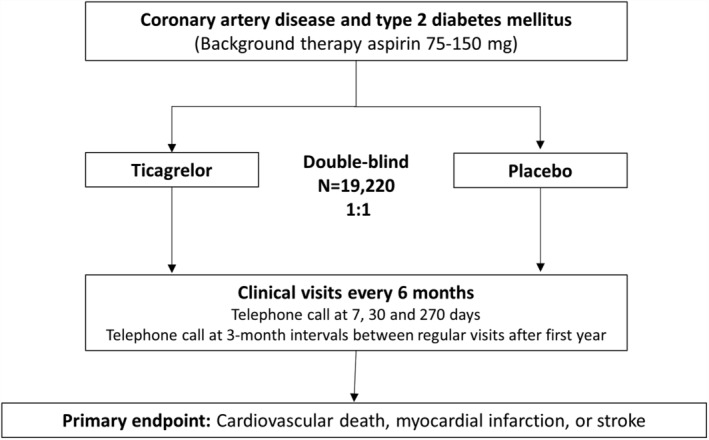
THEMIS study design. Data as of Feb 8, 2019

THEMIS (NCT01991795) is a Phase 3b randomized, double‐blinded, placebo‐controlled trial of ticagrelor vs placebo, on top of low dose aspirin (75‐150 mg) unless contraindicated or not tolerated. The primary hypothesis of the trial is that twice daily ticagrelor when added to aspirin will reduce the risk of CV death, MI, or stroke in patients with DM and stable coronary artery disease. Patients were initially randomized to 90 mg twice daily of ticagrelor or matching placebo (Figure 1). Partway through the trial, the dose in the ticagrelor arm was lowered to 60 mg twice daily to be consistent with updated product labeling of ticagrelor in response to external clinical trial data in patients with an MI more than 1 year previously.^14^ Specifically, in PEGASUS, the efficacy profiles of ticagrelor 90 mg twice daily and 60 mg twice daily administered with low‐dose ASA were similar to each other.^15^ The lower dose had a better tolerability profile with regard to dyspnea, less risk of bleeding, and led to fewer discontinuations from study drug. Patients with diabetes did not have a different efficacy and safety profile to that of the overall study population with respect to the two dosing strategies of ticagrelor.^14^ Hence, to use the lowest effective dose and taking the overall benefit‐risk into consideration, the dose in THEMIS was changed to ticagrelor 60 mg twice daily. A protocol amendment enabling a dose reduction was finalized in May 2015, resulting in that approximately 25% of the randomized patients started on ticagrelor 60 mg twice daily or matching placebo. Because the lower dose was introduced rather early in the study, it is expected that at end of the study, at least 75% of the exposure time will be on 60 mg twice daily. The first patient was randomized on February 10, 2014 and the last patient on May 24, 2016. A total of 1315 sites in 42 countries were involved in the study.

Patients ≥50 years with DM receiving anti‐diabetic medications for at least 6 months with stable coronary artery disease as determined by a history of previous percutaneous coronary intervention, coronary artery bypass grafting, or angiographic stenosis of ≥50% of at least one coronary artery were enrolled. Patients with known prior MI or stroke were excluded. Detailed inclusion and exclusion criteria are listed in Table 1. If a clinical indication arose, open‐label use of DAPT was allowed in the trial and patients came off blinded study drug for that duration.

**Table 1:** Inclusion and exclusion criteria

Inclusion criteria
Provide informed consent prior to any study specific procedures
Men or women ≥50 years of age
Diagnosed with T2DM defined by ongoing glucose lowering drug treatment prescribed by a physician for treatment of T2DM since at least 6 months prior to 1st visit
At high risk of CV events, defined as a history of percutaneous coronary intervention or coronary artery bypass graft or angiographic evidence of ≥50% stenosis of at least one coronary artery
**Exclusion criteria**
Previous MI^a^ except for definite secondary MI (eg, due to coronary revascularization procedure, profound hypotension, hypertensive emergency, tachycardia, or profound anemia)
Previous stroke (TIA is not included in the stroke definition)
Planned use of ADP receptor antagonists (eg, clopidogrel, ticlopidine, prasugrel), dipyridamole, or cilostazol. Planned use of aspirin >150 mg once a day
Planned coronary, cerebrovascular, or peripheral artery revascularization
Anticipated concomitant oral or intravenous therapy with strong CYP3A4 inhibitors (ketoconazole, itraconazole, voriconazole, telithromycin, clarithromycin, nefazodone, ritonavir, saquinavir, nelfinavir, indinavir, atazanavir) or CYP3A4 substrates with narrow therapeutic indices (quinidine, simvastatin >40 mg daily or lovastatin >40 mg daily) which cannot be stopped
Need for chronic oral anticoagulant therapy or chronic low‐molecular‐weight heparin (at venous thrombosis treatment not prophylaxis doses)
Known bleeding diathesis or coagulation disorder, or uncontrolled hypertension (defined as a systolic BP ≥180 mm Hg and/or diastolic BP ≥100 mmHg)
History of previous intracerebral bleed at any time, gastrointestinal bleed within 6 months prior to randomization, or major surgery within 30 days prior to randomization
Increased risk of bradycardic events (eg, known sick sinus syndrome, second or third degree AV block, or previous documented syncope suspected to be due to bradycardia) unless treated with a pacemaker
Known severe liver disease
Renal failure requiring dialysis
Pregnancy or lactation, and women of child‐bearing potential not using reliable contraception
Concern for inability to comply with study procedures and/or follow‐up, or any conditions (judged by the investigator) that may render the patient unable to complete the study
Any condition judged by the investigator that make participation unsafe or unsuitable, or any condition outside the atherothrombotic study area with a life expectancy <2 years
Participation in another clinical study with an investigational product within 28 days prior to enrolment, or previous randomization to an investigational product in another ongoing clinical study. Participation in any previous study with ticagrelor. Previous randomization in the present study
Involvement in the planning/conduct of the study

Abbreviations: ADP, adenosine diphosphate; BP, blood pressure; CV, cardiovascular; CYP, cytochrome P450; MI, myocardial infarction; T2DM, type 2 diabetes mellitus; TIA, transient ischemic attack.

a Previous MI is a documented hospitalization with a final diagnosis of spontaneous MI.

The primary efficacy endpoint is a composite of CV death, myocardial infarction, or stroke. The primary safety endpoint is Thrombolysis in Myocardial Infarction major bleeding. Table 2 provides further details about the pre‐specified hierarchical testing of secondary endpoints. Appendix S1, Supporting Information provides the definitions. All endpoints were adjudicated by a blinded academic clinical endpoint committee. Partway through the trial, given an increasing appreciation of the importance of peripheral artery ischemic endpoints, these were also adjudicated by a blinded academic clinical endpoint committee.^15^, ^16^, ^17^ To ensure data integrity, there was a firewall maintained between the clinical endpoint committee (CEC) and the data monitoring committee (DMC). Specifically, the DMC statistical data analysis center (SDAC), handling all unblinded study data, was located at Duke Clinical Research Institute (DCRI).

**Table 2:** Primary and secondary efficacy variables

Primary efficacy variable	Secondary efficacy variables (in hierarchical order)
Time from randomization to the first occurrence of any event from the composite of CV death, MI, or stroke (ischemic, hemorrhagic, or unknown etiology)	Time from randomization to death of CV cause
	Time from randomization to the first occurrence of MI
	Time from randomization to the first occurrence of ischemic stroke
	Time from randomization to death of any cause

Abbreviations: CV, cardiovascular; MI, myocardial infarction.

The primary efficacy endpoint will be tested at a 4.96% significance level (two‐sided), adjusted for 1 planned efficacy interim analysis with family‐wise error controlled at 5%. The one planned efficacy interim analysis occurred on the 29th of March 2017. The recommendation from the DMC was to continue the study according to the protocol. The estimated annual event rate in the placebo arm was 2.5%. An effect size of 16% relative risk reduction was hypothesized, with 1385 primary endpoint events needed to provide 90% power. This resulted in an estimated sample size of 19 000 patients, randomized in a 1:1 ratio, with an average follow‐up time of 40 months (maximum 58 months). Even with the ticagrelor dosage switch, a power of 90% in the study is maintained, as the main analysis is based on randomization to ticagrelor irrespective of dose.

There are no sub‐studies planned, but predefined subgroup analyses will explore efficacy and safety according to baseline characteristics, such as revascularization history, single vs multivessel coronary artery disease, duration of diabetes, glycemic control, anti‐diabetic medications, age groups, and renal function.

## 3 RESULTS

**Figure 2:**
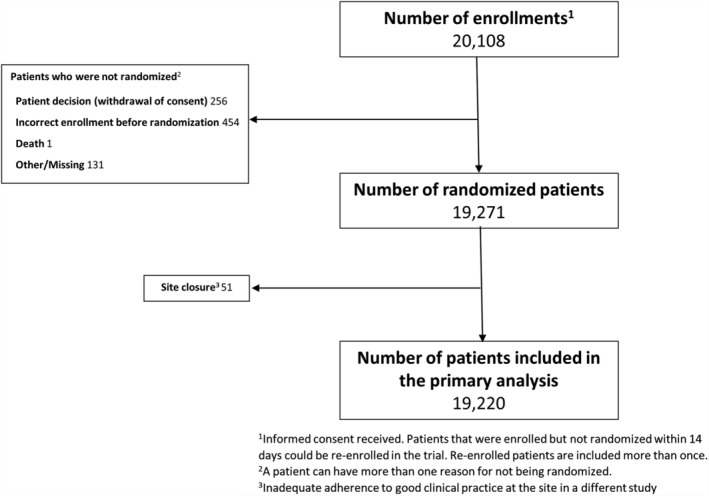
THEMIS study flow diagram. Data as of Feb 8, 2019

A total of 20 108 patients were enrolled. Of these, 19 271 were randomized, and because of closure of a single site secondary for inadequate adherence to good clinical practice in a different trial, 19 220 patients are expected to be available for analysis (Figure 2).

The baseline characteristics and medications are described in Table 3. The randomized population had a median age of 66 years and 31.4% were women. As expected in patients with DM, there was a high prevalence of concomitant hypertension and dyslipidemia. The prevalence of multivessel coronary artery disease was 62.1%, although 20.2% had no prior revascularization. The median duration of DM was 10.0 years, the hemoglobin A1c at baseline was 7.1%, and 28.7% of the patients were treated with insulin. At baseline, the population was very well‐treated, with 89.8% on statins and 78.6% on an angiotensin‐converting enzyme inhibitor or angiotensin receptor blocker.

**Table 3:** Baseline characteristics. (not final data; data as of Feb 8, 2019)

Characteristic	Randomized patients (N = 19 220)^a^
Age (years), median (IQR)	66.0 (61.0‐72.0)
Male, n (%)	13 189 (68.6)
BMI (kg/m^2^), median (IQR)	29.0 (26.0‐32.7)
Current smoker, n (%)	2094 (10.9)
Race, n (%)	
Asian	4406 (22.9)
Black or African American	403 (2.1)
Other	715 (3.7)
White	13 696 (71.3)
Geographic region, n (%)	
Asia and Australia	4288 (22.3)
Central and South America	2169 (11.3)
Europe, Middle East, and South Africa	9768 (50.8)
North America	2995 (15.6)
Disease history	
Hypertension, n (%)	17 776 (92.5)
Dyslipidemia, n (%)	16 753 (87.2)
Angina pectoris, n (%)	10 801 (56.2)
Multi‐vessel coronary artery disease (>1 vessel), n (%)	11 935 (62.1)
Revascularization status, n (%)	
Previous PCI only	9808 (51.0)
Previous CABG only	4191 (21.8)
Previous PCI and CABG	1346 (7.0)
No previous revascularization^b^	3875 (20.2)
Time since most recent PCI (years), median (IQR)	3.3 (1.5‐6.6)
Time since most recent CABG (years), median (IQR)	4.3 (1.5‐9.2)
History of peripheral artery disease, n (%)	1687 (8.8)
History of poly‐vascular disease^c^, n (%)	2579 (13.4)
Duration of diabetes (years), median (IQR)	10.0 (5.0‐16.0)
History of any diabetes complications^d^, n (%)	4910 (25.5)
HbA1c at baseline (%), median (IQR)	7.1 (6.4‐8.1)
eGFR (MDRD) at baseline (mL/min/1.73 m^2^), median (IQR)	75.0 (60.5‐89.6)
Medication use at baseline^e^	
Aspirin, n (%)	19 104 (99.4)
Aspirin dose (mg), median (IQR)	100 (80‐100)
Statin, n (%)	17 266 (89.8)
Proton pump inhibitor, n (%)	4901 (25.5)
ACE‐inhibitor or ARB, n (%)	15 113 (78.6)
ACE‐inhibitor	8145 (42.4)
ARB	7211 (37.5)
Beta‐blocker, n (%)	14 192 (73.8)
Insulin, n (%)	5508 (28.7)
Any diabetes medications, n (%)	19 156 (99.7)
1	8609 (44.8)
2	6911 (36.0)
3	2892 (15.0)
>3	744 (3.9)

Abbreviations: ACE, angiotensin‐converting enzyme; ARB, angiotensin receptor blocker; BMI, body mass index; CABG, coronary artery bypass grafting; CAD, coronary artery disease; eGFR, estimated glomerular filtration rate; HbA1c, glycated hemoglobin; IQR, interquartile range; MDRD, modification of diet in renal disease; PCI, percutaneous coronary intervention; PAD, peripheral artery disease.

a N is the total number of randomized patients, patients that have been randomized more than once are only included according to their first randomization. Patients that are randomized but will not be included in the primary analysis are not included in this table;

b significant stenosis on coronary angiography but no revascularization;

c Defined as arterial obstructive disease involving at least 2 vascular beds where vascular bed involvement is characterized by either 1) CAD (defined as CAD, PCI or CABG), 2) PAD, 3) carotid artery stenosis or cerebral revascularization;

d Defined as at least one of retinopathy, autonomic neuropathy, peripheral neuropathy, and nephropathy;

e Medications used within 30 days of randomization, aspirin use is captured on day of randomization.

## 4 DISCUSSION

The THEMIS trial was designed to address an important question in the management of patients with DM and stable coronary artery disease but no prior MI—does intensification of the antiplatelet regimen beyond aspirin reduce the risk of CV events? The trial is fully enrolled and well‐powered to detect moderate relative risk reductions in the overall population studied. The sample size will hopefully allow examination of clinically logical and well‐defined subgroups even if the overall trial does not meet its primary endpoint or shows marginal net clinical benefit. The evidence from THEMIS will complement that of several other trials which assessed the value of DAPT in patients with acute coronary syndromes or prior myocardial infarction.^2^, ^7^, ^11^, ^12^, ^15^

When added to aspirin, ticagrelor specifically provides superior benefit compared with clopidogrel in patients with acute coronary syndromes^7^ and compared with placebo in high‐risk patients with prior myocardial infarction.^15^ There is lack of clear benefit of ticagrelor monotherapy, compared with clopidogrel in peripheral artery disease^18^ and compared with aspirin in ischemic stroke.^19^ In the context of patients undergoing coronary stenting, there was no clear benefit to a strategy of DAPT for a month followed by ticagrelor monotherapy over a more conventional strategy of DAPT for a year followed by aspirin monotherapy, although additional study is ongoing in this regard.^20^, ^21^, ^22^

There is evidence that platelet aggregation is enhanced in patients with DM compared with those without DM and that aspirin may have reduced efficacy in DM.^23^ This appears to be because of several factors, including accelerated platelet turnover. Indeed, there is some evidence that twice daily administration of aspirin is more effective at inhibition of generation of thromboxane than once a day administration.^24^, ^25^ In addition, enteric coated aspirin is widely used and appears to be somewhat less effective than non‐enteric coated aspirin in pharmacodynamic and pharmacokinetic analyses.^26^ Recent data suggest that aspirin has only a very modest effect for primary prevention CV events in patients with DM.^27^, ^28^, ^29^

Similarly, there is also evidence of reduced pharmacodynamic and pharmacokinetic efficacy of clopidogrel among patients with DM, in part because of less efficient metabolism of clopidogrel as a prodrug.^30^, ^31^, ^32^, ^33^ However, the clinical implications of these observations are not entirely clear since clopidogrel did not appear less effective in patients with DM in a large outcome trial of clopidogrel vs aspirin.^34^

With more effective oral ADP receptor antagonists than clopidogrel, such as prasugrel or ticagrelor, there is no evidence of reduced efficacy in patients with DM who present with acute coronary syndromes or long‐term post‐MI.^14^, ^35^, ^36^ In particular, some of the concerns regarding the efficacy of aspirin are related to short plasma residence time of the drug, while there is accelerated platelet generation during the 24‐hour cycle. As ticagrelor is a reversible inhibitor of the P2Y_12_ receptor, it is given twice daily. This results in persistent plasma levels that may be able to antagonize the P2Y_12_ receptor in newly formed platelets around the entire circadian cycle. These observations led to the hypothesis of the THEMIS trial that DAPT with ticagrelor plus aspirin would be superior to aspirin alone even in DM patients with stable coronary artery disease.^14^

The baseline characteristics of the THEMIS population suggest that it is very representative of daily practice in the care of DM patients. Thus, the results of THEMIS should be quite generalizable to the large number of patients with DM and stable coronary artery disease worldwide. Furthermore, the background medical therapy, such as statin use, is excellent, and therefore any observed benefits would truly be an incremental advance.

Furthermore, THEMIS is the largest randomized trial of patients with DM performed to date. Beyond the randomized question being addressed, the THEMIS database will allow several important hypotheses to be tested in observational analyses using a large, well‐characterized population of patients with coronary artery disease and DM.

Limitations include the lowering of dose that occurred partway through the trial from ticagrelor 90 mg twice daily to ticagrelor 60 mg twice daily, which reduces the statistical power to examine either dose alone. Given the overall similar efficacy but trend towards more bleeding and less tolerability of ticagrelor 90 mg twice daily compared with ticagrelor 60 mg twice daily in PEGASUS, the lowering of the dose in THEMIS should impact efficacy less but could overestimate the overall bleeding risk, which is a conservative approach.^37^, ^38^, ^39^ Multiple subgroup analyses have been prespecified, such as patients with multivessel coronary artery disease and prior percutaneous or surgical revascularization, but despite its size, the trial may not be well powered for subgroup analysis. Some peripheral ischemic events may not have been captured, as adjudication of these endpoints were added partway through the trial, though the trial was still blinded when this occurred, thus any assessment should be unbiased.

## 5 CONCLUSION

Approaches to reduce CV morbidity further in patients with DM with stable atherosclerosis are urgently needed. More intense antiplatelet therapy is a promising approach. The THEMIS trial is assessing whether DAPT with ticagrelor and low‐dose aspirin provides a significant reduction in ischemic events with an acceptable increase in bleeding. If the trial is positive overall, it will change the treatment paradigm for patients with diabetes. If the trial is clearly negative overall and in all major subgroups, it will provide a clear risk level below which more intense antiplatelet therapy is not indicated. Thus, irrespective of the exact results, THEMIS should greatly refine our understanding of the role of DAPT in patients across the atherothrombotic spectrum.

## CONFLICT OF INTEREST

Dr. Deepak L. Bhatt discloses the following relationships: Advisory Board: Cardax, Elsevier Practice Update Cardiology, Medscape Cardiology, Regado Biosciences; Board of Directors: Boston VA Research Institute, Society of Cardiovascular Patient Care, TobeSoft; Chair: American Heart Association Quality Oversight Committee; Data Monitoring Committees: Baim Institute for Clinical Research (formerly Harvard Clinical Research Institute, for the PORTICO trial, funded by St. Jude Medical, now Abbott), Cleveland Clinic (including for the ExCEED trial, funded by Edwards), Duke Clinical Research Institute, Mayo Clinic, Mount Sinai School of Medicine (for the ENVISAGE trial, funded by Daiichi Sankyo), Population Health Research Institute; Honoraria: American College of Cardiology (Senior Associate Editor, Clinical Trials and News, ACC.org; Vice‐Chair, ACC Accreditation Committee), Baim Institute for Clinical Research (formerly Harvard Clinical Research Institute; RE‐DUAL PCI clinical trial steering committee funded by Boehringer Ingelheim), Belvoir Publications (Editor in Chief, Harvard Heart Letter), Duke Clinical Research Institute (clinical trial steering committees), HMP Global (Editor in Chief, Journal of Invasive Cardiology), Journal of the American College of Cardiology (Guest Editor; Associate Editor), Population Health Research Institute (for the COMPASS operations committee, publications committee, steering committee, and USA national co‐leader, funded by Bayer), Slack Publications (Chief Medical Editor, Cardiology Today's Intervention), Society of Cardiovascular Patient Care (Secretary/Treasurer), WebMD (CME steering committees); Other: Clinical Cardiology (Deputy Editor), NCDR‐ACTION Registry Steering Committee (Chair), VA CART Research and Publications Committee (Chair); Research Funding: Abbott, Amarin, Amgen, AstraZeneca (including for his role as co‐Chair of THEMIS), Bayer, Boehringer Ingelheim, Bristol‐Myers Squibb, Chiesi, Eisai, Ethicon, Forest Laboratories, Idorsia, Ironwood, Ischemix, Lilly, Medtronic, PhaseBio, Pfizer, Regeneron, Roche, Sanofi Aventis, Synaptic, The Medicines Company; Royalties: Elsevier (Editor, Cardiovascular Intervention: A Companion to Braun wald's Heart Disease); Site Co‐Investigator: Biotronik, Boston Scientific, St. Jude Medical (now Abbott), Svelte; Trustee: American College of Cardiology; Unfunded Research: FlowCo, Fractyl, Merck, Novo Nordisk, PLx Pharma, Takeda. Dr Kim Fox receives fees, honoraria and/or travel expenses from Servier, TauRx, CellAegis, Celixir and Broadview Ventures. He is a director of Vesalius Trials Ltd. Dr Harrington discloses research grants/contracts from the National Heart Lung and Blood Institute, Duke, AstraZeneca, CSL‐Behring, Glaxo Smith Kline, Merck, Portola, Regado, Sanofi‐Aventis, and The Medicines Company, and consulting/advisory for Adverse Events, Amgen, Element Science, Gilead, Merck, MyoKardia, The Medicines Company, VidaHealth, and WebMD. Dr. Lawrence Leiter discloses the following relationships: research grants from Amgen, AstraZeneca, Bayer, Boehringer Ingelheim, Eli Lilly, Esperion, GSK, Kowa, Novo Nordisk, Sanofi, The Medicines Company; speaking or consulting fees from Amgen, AstraZeneca, Boehringer Ingelheim, Eli Lilly, Merck, Novartis, Novo Nordisk, Sanofi. Dr. Shamir Mehta discloses the following relationships: Institutional research grants from Abbott Vascular, AstraZeneca, Boston Scientific and Sanofi and consulting fees from AstraZeneca and Sanofi. Dr Tabassome Simon discloses the following relationships: research grants from AstraZeneca, Daiichi‐Sankyo, Eli‐Lilly, GSK, MSD, Novartis, Sanofi, speaking or consulting fees from AstraZeneca, BMS, Novartis Sanofi. Dr Claes Held discloses the following relationships: Institutional research grants from AstraZeneca, GlaxoSmith Kline, BristolMyers Squibb, Pfizer and Merck. Advisory board ‐ AstraZeneca, Bayer, Boehringer Ingelheim, Idorsia and Coala Life. Speaking or consulting fees from AstraZeneca, Bayer. Dr. Ph. Gabriel Steg discloses the following relationships: research grants from Amarin, Bayer, Merck, Sanofi, and Servier; speaking or consulting fees from Amarin, Amgen, AstraZeneca, Bayer/Janssen, Boehringer‐Ingelheim, Bristol‐Myers Squibb, Idorsia, Lilly, Merck, Novartis, Novo‐Nordisk, Pfizer, Regeneron, Sanofi, Servier. Dr Anders Himmelmann, Dr Wilhelm Ridderstråle, and Marielle Andersson report being employees of AstraZeneca.

## Supporting information


**Appendix S1.** Supplementary Appendix to Bhatt et al., Rationale, Design, and Baseline Characteristics of THEMIS: Effect of Ticagrelor on Health Outcomes in Diabetes Mellitus Patients Intervention Study

## Appendix

**Executive Committee**

Deepak L. Bhatt MD MPH (co‐Chair and co‐Principal Investigator), Kim Fox MD, Robert A. Harrington MD, Lawrence A. Leiter MD, Shamir R. Mehta MD MSc, Tabassome Simon, MD, PhD, Marielle Andersson MSc, Anders Himmelmann MD, PhD, Ph. Gabriel Steg MD (co‐Chair and co‐Principal Investigator).

**Steering Committee**

Rafael Diaz (Argentina), John Amerena (Australia), Kurt Huber (Austria), Peter Sinnaeve (Belgium), José Carlos Nicolau (Brazil), José Francisco Kerr Saraiva (Brazil), Ivo Petrov (Bulgaria), Lawrence A. Leiter (Canada), Shamir R. Mehta (Canada), Ramón Corbalán (Chile), Junbo Ge (China), Qiang Zhao (China), Rodrigo Botero (Colombia), Petr Widimský (Czech Republic), Steen Dalby Kristensen (Denmark), Juha Hartikainen (Finland), Nicolas Danchin (France), Harald Darius (Germany), Tse Hung Fat (Hong Kong), Robert Gabor Kiss (Hungary), Prem Pais (India), Eli Lev (Israel), Leonardo De Luca (Italy), Shinya Goto (Japan), Gabriel Arturo Ramos López (Mexico), Jan Hein Cornel (Netherlands), Frederic Kontny (Norway), Felix Medina (Peru), Noe Babilonia (Philippines), Grzegorz Opolski (Poland), Dragos Vinereanu (Romania), Dmitry Zateyshchikov (Russia), Mikhail Ruda (Russia), Omer Elamin (Saudi Arabia), František Kovář (Slovakia), Anthony John Dalby (South Africa), Myung Ho Jeong (South Korea), Héctor Bueno (Spain), Stefan James (Sweden), Chern‐En Chiang (Taiwan), Damras Tresukosol (Thailand), Zeki Ongen (Turkey), Kausik Ray (UK), Alexander Parkhomenko (Ukraine), Darren McGuire (USA), Mikhail Kosiborod (USA), Tuan Quang Nguyen (Vietnam).

**Data Monitoring Committee**

Lars Wallentin MD, PhD (Chair), Keith A. A. Fox MB ChB, John W. Eikelboom MBBS, Jaakko Tuomilehto MD, Kerry L. Lee PhD; Hussein R. al‐Khalidi PhD and Stephen J. Ellis PhD (non‐voting independent statisticians, DCRI).

**Clinical Endpoint Committee**

Uppsala Clinical Research Centers members

Claes Held MD, PhD (CEC Chair), Emil Hagström MD, PhD (CEC co‐chair), Pernilla Holmgren (CEC Project Manager), Ulrika Heldestad (CEC Coordinator Consultant), Theresa Hallberg (CEC Coordinator), Karin Renlund Grausne (CEC coordinator), Cristina Alm (CEC monitor), Åsa Michelgård Palmquist (CEC monitor), Camilla Svanberg (CEC administrator).

Colorado Prevention Center (CPC) members

Warren H. Capell MD (CEC Co‐chair), Mark R. Nehler MD (CEC Co‐chair), William R. Hiatt MD (Senior Scientific Lead), Marc P. Bonaca MD (Senior Scientific Lead), Stacey Houser (CEC Operations Manager), Susie Bachler (CEC Coordinator), Nicole Jaeger (Executive Project Manager).

### AstraZeneca

Marielle Andersson MSc (Statistician), Maria Aunes MD (Safety physician), Åsa Brusehed (Project management lead), Jersey Chen MD (Study physician), Björn Dahlöf (Programming), Jitka Doležalová (Site Management and monitoring), Maciej Domzol (Study leader), Magdalena Findley (Study management), Anders Himmelmann MD, PhD (Clinical lead physician), Niclas Holmberg (Programming), Marianne Jahreskog RN (Safety scientist), Mikael Knutsson PhD (Statistician), Jakub Kruszewski (Enablement), Maria Leonsson‐Zachrisson MD, PhD (Senior physician), Wilhelm Ridderstråle MD, PhD (Study physician), Maj Stark (Clinical Development), Elin Winder (Data management).

**Countries Participating in THEMIS**

Argentina, Australia, Austria, Belgium, Brazil, Bulgaria, Canada, Chile, China, Colombia, Czech Republic, Denmark, Finland, France, Germany, UK, Hong Kong, Hungary, India, Israel, Italy, Japan, Mexico, Netherlands, Norway, Peru, Philippines, Poland, Republic of Korea, Romania, Russia, Saudi Arabia, Slovakia, South Africa, Spain, Sweden, Taiwan, Thailand, Turkey, Ukraine, USA, Vietnam.
