# Supplementary material for: Rationale, design and baseline characteristics of the effect of ticagrelor on health outcomes in diabetes mellitus patients Intervention study
Source: Clin Cardiol. 2019 Apr 9;42(5):498–505. doi: 10.1002/clc.23164 (PMC6522985; doi:10.1002/clc.23164)
Supplement: Supplementary file 1 — Appendix S1. Supplementary Appendix to Bhatt et al., Rationale, Design, and Baseline Characteristics of THEMIS: Effect of Ticagrelor on Health Outcomes in Diabetes Mellitus Patients Intervention Study [file CLC-42-498-s001.docx]

**Supplementary Appendix**

Supplement to: **Rationale, Design, and Baseline Characteristics of THEMIS: Effect of Ticagrelor on Health Outcomes in Diabetes Mellitus Patients Intervention Study**

Bhatt DL, Fox K, Harrington RA et al. Clinical Cardiology. 2019:xx

**Contents**

[ENDPOINT DEFINITIONS 3](#_Toc1403535)

[**ISCHEMIC EVENTS** 3](#_Toc1403536)

[Death 3](#_Toc1403537)

[Definition of Myocardial Infarction 5](#_Toc1403540)

[Definition of Unstable Angina 6](#_Toc1403544)

[Definition of Urgent Coronary Revascularization 7](#_Toc1403545)

[Definition of Stent Thrombosis 7](#_Toc1403546)

[Definition of Stroke 8](#_Toc1403547)

[Definition of Transient Ischemic Attack 9](#_Toc1403550)

[Intracranial hemorrhage (ICH) 9](#_Toc1403551)

[**BLEEDING EVENTS** 9](#_Toc1403553)

[TIMI Bleeding Criteria 10](#_Toc1403556)

[PLATO Bleeding Classification 11](#_Toc1403557)

[BARC Bleeding Definitions 11](#_Toc1403558)

#

# ENDPOINT DEFINITIONS

# ISCHEMIC EVENTS

## Death

All deaths reported post-randomization will be recorded and adjudicated. Deaths after patient withdrawal of consent will be recorded but not adjudicated.

Deaths will be sub-classified by CV and non-CV primary cause. CV death includes sudden cardiac death, death due to acute MI, death due to heart failure or cardiogenic shock, death due to a cerebrovascular event, death due to other CV causes (e.g., pulmonary embolism, aortic disease, CV intervention), and deaths for which there was no clearly documented non- CV cause (presumed CV death).

## Cardiovascular Death

Cardiovascular death includes sudden cardiac death, death due to acute myocardial infarction, death due to heart failure, death due to a cerebrovascular event, and death due to other cardiovascular causes, as follows:

1. **Sudden Cardiac Death**: refers to death that occurs unexpectedly and includes the following deaths:
2. Death witnessed and instantaneous without new or worsening symptoms
3. Death witnessed within 60 minutes of the onset of new or worsening cardiac symptoms
4. Death witnessed and attributed to an identified arrhythmia (e.g., captured on an electrocardiographic (ECG) recording or witnessed on a monitor by either a medic or paramedic)
5. Death after unsuccessful resuscitation from cardiac arrest or successfully resuscitated from cardiac arrest but who die within 24 hours without identification of a non-cardiac etiology
6. Death > 24hrs after a patient has been successfully resuscitated from cardiac arrest and without identification of a non-cardiovascular etiology
7. Unwitnessed death or other causes of death (information regarding the patient’s clinical status within the week preceding death should be provided if available)
8. **Death due to Acute Myocardial Infarction (MI**): refers to a death within 30 days after a myocardial infarction (MI) related to consequences seen immediately after the myocardial infarction, such as progressive congestive heart failure (CHF), inadequate cardiac output, or refractory arrhythmia. If these events occur after a “break” (e.g., a CHF and arrhythmia free period), they should be designated by the immediate cause. The acute MI should be verified by the diagnostic criteria outlined for acute MI (including autopsy findings showing recent MI or recent coronary thrombus) and there should be no conclusive evidence of another cause of death.

Sudden, unexpected cardiac death, involving cardiac arrest, often with symptoms suggestive of myocardial ischemia, and accompanied by presumably new ST elevation, or new LBBB and/or evidence of fresh thrombus by coronary angiography and/or at autopsy, but death occurring before blood samples could be obtained, or at a time before the appearance of cardiac biomarkers in the blood should be considered death due to acute myocardial infarction. Death resulting from a procedure to treat myocardial ischemia or to treat a complication resulting from myocardial infarction should also be considered death due to acute MI.

If death occurs before biochemical confirmation of myocardial necrosis can be obtained, adjudication should be based on clinical presentation and ECG evidence.

Death due to a myocardial infarction that occurs as a direct consequence of a cardiovascular investigation/procedure/operation will be classified as death due to other cardiovascular cause.

1. **Death due to Heart Failure or Cardiogenic Shock:** refers to death occurring in the context of clinically worsening symptoms and/or signs of heart failure not in the context of an acute MI and without evidence of another cause of death. New or worsening signs and/or symptoms of congestive heart failure (CHF) include any of the following:
2. New or increasing symptoms and/or signs of heart failure requiring the initiation of, or an increase in, treatment directed at heart failure or occurring in a patient already receiving maximal therapy for heart failure.
3. Heart failure symptoms or signs requiring continuous intravenous drug therapy or oxygen administration.
4. Confinement to bed predominantly due to heart failure symptoms.
5. Pulmonary edema sufficient to cause tachypnea and distress not occurring in the context of an acute myocardial infarction or as the consequence of an arrhythmia occurring in the absence of worsening heart failure
6. Cardiogenic shock not occurring in the context of an acute MI or as the consequence of an arrhythmia occurring in the absence of worsening heart failure.

Cardiogenic shock is defined as systolic blood pressure (SBP) < 90 mm Hg for greater than 1 hour, not responsive to fluid resuscitation and/or heart rate correction, and felt to be secondary to cardiac dysfunction and associated with at least one of the following signs of hypoperfusion:

1. Cool, clammy skin or
2. Oliguria (urine output < 30 mL/hour) or
3. Altered sensorium or
4. Cardiac index < 2.2 L/min/m^2^

Cardiogenic shock can also be defined as SBP ≥90 mm Hg as a result of positive inotropic or vasopressor agents alone and/or with mechanical support in less than 1 hour.

This category will include sudden death occurring during an admission for worsening heart failure.

1. **Death due to Cerebrovascular Event (intracranial hemorrhage or non-hemorrhagic stroke): r**efers to cerebrovascular event the sequelae of which lead to death, generally within 30 days. The cerebrovascular event should be verified by the diagnostic criteria outlined for cerebrovascular events (including autopsy findings) and there should be no conclusive evidence of another cause of death.
2. **Death due to Other Cardiovascular Causes:** death must be due to a fully documented cardiovascular cause not included in the above categories (e.g. pulmonary embolism, aortic disease (dissection or rupture), pericardial tamponade, or cardiovascular intervention).
3. **Presumed Cardiovascular Death:** All deaths not attributed to the categories of cardiovascular death and not attributed to a non-cardiovascular cause, are presumed cardiovascular deaths and as such are part of the cardiovascular mortality endpoint.

Of note, Coronary Heart Disease death will include the following categories; Sudden Cardiac Death, Death due to Acute MI, and the subset of Death due to other Cardiovascular Causes that are secondary to a coronary revascularization procedure.

## Non-Cardiovascular Death

Non-cardiovascular death is defined as any death not covered by cardiovascular death and falling into one of the following categories:

- Pulmonary failure
- Renal failure
- Gastrointestinal causes
- Hepatobiliary
- Pancreatic
- Infection (includes sepsis)
- Non-infectious (e.g. SIRS)
- Hemorrhage that is neither CV bleeding or stroke
- Non-CV procedure or surgery
- Trauma
- Suicide
- Non-prescription drug reaction or overdose
- Prescription drug reaction or overdose
- Neurological (non-cardiovascular)
- Malignancy
- Other, please specify.

## Definition of Myocardial Infarction

CEC will use the Third Universal MI definition (Thygesen et al 2012) as study specific MI criteria during adjudication.

## Criteria for Acute Myocardial Infarction

The term acute MI should be used when there is evidence of myocardial necrosis in a clinical setting consistent with acute myocardial ischemia. Under these conditions any one of the following criteria meets the diagnosis for MI:

1. Detection of a rise and/or fall of cardiac biomarker values (preferably cardiac troponin [cTn]) with at least one value above the 99th percentile upper reference limit (URL) and with at least one of the following:
   1. Symptoms of ischemia
   2. New or presumed new significant ST-segment–T wave (ST–T) changes or new left bundle branch block (LBBB)
   3. Development of pathological Q waves in the ECG
   4. Imaging evidence of new loss of viable myocardium or new regional wall motion abnormality
   5. Identification of an intracoronary thrombus by angiography or autopsy
2. Cardiac death with symptoms suggestive of myocardial ischemia and presumed new ischemic ECG changes or new LBBB, but death occurred before cardiac biomarkers were obtained, or before cardiac biomarker values would be increased
3. PCI-related MI is arbitrarily defined by elevation of cTn values (>5 x 99th percentile URL) in patients with normal baseline values (≤99th percentile URL) or a rise of cTn values >20% if the baseline values are elevated and are stable or falling. In addition, either (i) symptoms suggestive of myocardial ischemia or (ii) new ischemic ECG changes or (iii) angiographic findings consistent with a procedural complication, or (iv) imaging demonstration of new loss of viable myocardium or new regional wall motion abnormality are required
4. Stent thrombosis associated with MI when detected by coronary angiography or autopsy in the setting of myocardial ischemia and with a rise and/or fall of cardiac biomarker values with at least one value above the 99th percentile URL
5. CABG- related MI is arbitrarily defined by elevation of cardiac biomarker values (>10 x 99th percentile URL) in patients with normal baseline cTn values (≤99th percentile URL). In addition, either (i) new pathological Q waves or new LBBB, or (ii) angiographic documented new graft or new native coronary artery occlusion, or (iii) imaging evidence of new loss of viable myocardium or new regional wall motion abnormality.

## Criteria for Prior Myocardial Infarction

Any one of the following criteria meets the diagnosis for prior MI:

- Pathological Q waves with or without symptoms in the absence of non-ischemic causes
- Imaging evidence of a region of loss of viable myocardium that is thinned and fails to contract, in the absence of a non-ischemic cause
- Pathological findings of a prior MI

##

## Universal Classification of MI

The following classification will be used by the CEC for classification of MI and will not be captured in the eCRFs.

- **Type 1**: Spontaneous MI

Spontaneous myocardial infarction related to atherosclerotic plaque rupture, ulceration, fissuring, erosion, or dissection with resulting intraluminal thrombus in one or more of the coronary arteries leading to decreased myocardial blood flow or distal platelet emboli with ensuing myocyte necrosis. The patient may have underlying severe CAD but on occasion non-obstructive or no CAD

- **Type 2:** Myocardial infarction secondary to an ischemic imbalance

In instances of myocardial injury with necrosis where a condition other than CAD contributes to an imbalance between myocardial oxygen supply and/or demand, eg. coronary endothelial dysfunction, coronary artery spasm, coronary embolism, tachy-/brady-arrhythmias, anemia, respiratory failure, hypotension, and hypertension with or without left ventricular hypertrophy

- **Type** **3**: Myocardial infarction resulting in death when biomarker values are unavailable

Cardiac death with symptoms suggestive of myocardial ischemia and presumed new ischemic ECG changes or new LBBB, but death occurring before blood samples could be obtained, before cardiac biomarker could rise, or in rare cases cardiac biomarkers were not collected

- **Type** **4a**: Myocardial infarction related to percutaneous coronary intervention (PCI). Myocardial infarction associated with PCI is arbitrarily defined by elevation of cTn values >5 x 99th percentile URL in patients with normal baseline values (<99th percentile URL) or a rise of cTn values >20% if the baseline values are elevated and are stable or falling. In addition, either (i) symptoms suggestive of myocardial ischemia, or (ii) new ischemic ECG changes or new LBBB, or (iii) angiographic loss of patency of a major coronary artery or a side branch or persistent slow- or no-flow or embolization, or (iv) imaging demonstration of new loss of viable myocardium or new regional wall motion abnormality are required
- **Type** **4b:** Myocardial infarction related to stent thrombosis

Myocardial infarction associated with stent thrombosis is detected by coronary angiography or autopsy in the setting of myocardial ischemia and with a rise and/ or fall of cardiac biomarkers values with at least one value above the 99th percentile URL

- **Type 5**: Myocardial infarction related to coronary artery bypass grafting (CABG)

Myocardial infarction associated with CABG is arbitrarily defined by elevation of cardiac biomarker values >10 x 99th percentile URL in patients with normal baseline cTn values (<99th percentile URL). In addition, either (i) new pathological Q waves or new LBBB, or (ii) angiographic documented new graft or new native coronary artery occlusion, or (iii) imaging evidence of new loss of viable myocardium or new regional wall motion abnormality.

## Definition of Unstable Angina

The diagnosis of unstable angina will require ischemic chest pain (or equivalent) at rest ≥10 minutes in duration considered to be myocardial ischemia upon final diagnosis *and* prompting hospitalization within 24 hours of the most recent symptoms, *and* without elevation in cardiac biomarkers of necrosis, *and* the presence of objective evidence of ischemia as defined by at least 1 of the following criteria:

1. New or worsening ST or T wave changes in ≥2 anatomically contiguous leads on a resting ECG (in the absence of LVH and LBBB):
   1. transient (<20 minutes) ST elevation at the J point ≥ 0.2 mV in men (> 0.25 mV in men < 40 years old) or ≥ 0.15 mV in women in leads V2-V3 and/or ≥ 0.1 mV in other leads, or
   2. horizontal or down-sloping ST depression ≥ 0.10 mV, or
   3. T-wave inversion ≥ 0.2 mV
2. Definite evidence of myocardial ischemia on myocardial scintigraphy (clear reversible perfusion defect), stress echocardiography (reversible wall motion abnormality), or MRI (myocardial perfusion deficit under pharmacologic stress) that is believed to be responsible for the myocardial ischemic symptoms/signs.
3. Angiographic evidence of ≥ 70% lesion and/or thrombus in an epicardial coronary artery that is believed to be responsible for the myocardial ischemic symptoms/signs.

## Definition of Urgent Coronary Revascularization

Urgent revascularization is not a separate endpoint but a subcategory for CIE (MI and UA). The diagnosis of urgent coronary revascularization requires both of the two following criteria are met:

1. Ischemic chest pain (or equivalent) at rest ≥ 10 minutes in duration or repeated episodes at rest lasting ≥5 minutes considered to be myocardial ischemia upon final diagnosis, and
2. Prompting hospitalization and percutaneous coronary revascularization within 7 days of the symptoms or surgical coronary revascularization within 14 days of symptoms.

Coronary revascularization events not meeting criteria below for “urgent” will be classified as non-urgent coronary revascularization.

## Definition of Stent Thrombosis

Stent thrombosis will be classified as per the Academic Research Consortium Definition (Cutlip DE et al. Circulation. 2007;115:2344-51).

**Definite Stent Thrombosis** – is considered to have occurred by either angiographic or pathological confirmation.

1. The presence of thrombus that originates in the stent or in the segment 5 mm proximal or distal to the stent and presence of at least 1 of the following criteria within a 48-hour window (The incidental angiographic documentation of stent occlusion in the absence of clinical signs or symptoms is not considered a confirmed stent thrombosis silent occlusion):
   1. Acute onset of ischemic symptoms at rest
   2. New ischemic ECG changes that suggest acute ischemia
   3. Typical rise and fall in cardiac biomarkers that represent a spontaneous MI
   4. Non-occlusive Thrombus: Intracoronary thrombus defined as a (spheric, ovoid, or irregular) noncalcified filling defect or lucency surrounded by contrast material (on 3 sides or within a coronary stenosis) seen in multiple projections, or persistence of contrast material within the lumen, or visible embolization of intraluminal material downstream.
   5. Occlusive Thrombus: TIMI 0 or TIMI 1 intrastent or proximal to a stent up to the most adjacent proximal side branch or main branch (if originates from the side branch)
2. Evidence of recent thrombus within the stent determined at autopsy or via examination of tissue retrieved following thrombectomy.

**Probable Stent Thrombosis** – Clinical definition of probable stent thrombosis is considered to have occurred after intracoronary stenting in the following cases:

1. Any unexplained death within the first 30 days.
2. Irrespective of the time after the index procedure, any MI that is related to documented acute ischemia in the territory of the implanted stent without angiographic confirmation of stent thrombosis and in the absence of any other obvious cause

**Possible Stent Thrombosis** – Clinical definition of possible stent thrombosis is considered to have occurred with any unexplained death from 30 days after intracoronary stenting until end of trial follow-up.

## Definition of Stroke

Stroke is defined as an acute episode of neurologic dysfunction attributed to a central nervous system vascular cause.

Stroke should be documented by imaging (eg, CT scan or magnetic resonance imaging [MRI] scan) showing an area of acute infarction compatible with the neurologic symptoms. Evidence obtained from autopsy can also confirm the diagnosis.

For an event to qualify as a stroke, ALL of the following 4 criteria need to be fulfilled:

1. Rapid onset of a focal/global neurological deficit with at least one of the following:
   1. Change in level of consciousness
   2. Change in Modified Rankin Scale
   3. Hemiplegia
   4. Hemiparesis
   5. Numbness or sensory loss affecting one side of the body
   6. Dysphasia/Aphasia
   7. Hemianopia (loss of half of the field of vision of one or both eyes)
   8. Amaurosis fugax (transient complete/partial loss of vision of one eye)
   9. Other new neurological sign(s)/symptom(s) consistent with stroke

(If the acute focal signs represent a worsening of a previous deficit, these signs must have either (1) persisted for more than one week, or (2) persisted for more than 24 hours and were accompanied by an appropriate new CT or MRI finding.

1. Duration of a focal/global neurological deficit ≥ 24 hours OR < 24 hours if this is because of at least one of the following therapeutic interventions:
2. Pharmacologic (i.e., thrombolytic drug administration)
3. Non-pharmacologic (i.e., neurointerventional procedure (e.g. intracranial angioplasty)) or
4. Available brain imaging clearly documents a new hemorrhage or infarct or
5. The neurological deficit results in death
6. No other readily identifiable non-stroke cause for the clinical presentation (e.g., brain tumor, trauma, infection, hypoglycemia, peripheral lesion)
7. Confirmation of the diagnosis by at least one of the following:
8. Internal medicine, neurology or neurosurgical specialist
9. Brain imaging procedure (at least one of the following):
10. CT scan
11. MRI scan
12. Cerebral vessel angiography
13. Lumbar puncture (i.e. spinal fluid analysis diagnostic of intracranial hemorrhage)

**Stroke will be sub classified, when possible, as either:**

## Primary Ischemic Stroke

Ischemic stroke is defined as an infarction of the central nervous system tissue that results from a thrombus or embolus impairing central nervous system perfusion (and not primarily due to hemorrhage). Hemorrhage may be a consequence of ischemic stroke. In this situation, the stroke should be recorded as an ischemic stroke with hemorrhagic transformation rather than a hemorrhagic stroke event.

## Primary Hemorrhagic Stroke

Primary hemorrhagic stroke is defined as an acute episode of focal or global cerebral or spinal dysfunction with a documented cause of intracranial hemorrhage on imaging (e.g., computed tomography (CT) scan or magnetic resonance imaging (MRI) scan) either in the cerebral parenchyma, or subarachnoid space. Evidence of hemorrhagic stroke obtained from lumbar puncture, neurosurgery, or autopsy can also confirm the diagnosis.

Microhemorrhages (<10 mm) evident only on MRI are not considered to be a hemorrhagic stroke. Subdural and epidural bleeding will be considered intracranial hemorrhage, but not strokes (see below).

## Definition of Transient Ischemic Attack

TIA is defined as a transient episode < 24 hours of neurological dysfunction caused by focal brain, spinal cord, or retinal ischemia, without acute infarction. Patients are required to be hospitalized within 48 hours of their most recent neurologic symptoms.

## Intracranial Hemorrhage (ICH)

ICH includes all bleeding events (excluding microhemorrhages (<10 mm) evident only on gradient echo MRI) that occur within the cranium. ICH will be categorized as follows:

Location (based on imaging study): intraparenchymal, intraventricular, subarachnoid, subdural, epidural, and other. The first 3 categories will also be classified as hemorrhagic strokes if they occurred outside the setting of trauma.

Precipitant (based on history): non-traumatic and traumatic.

## Unknown/No Imaging Performed

The type of stroke could not be determined by imaging or other means (from lumbar puncture, neurosurgery, or autopsy) but is judged to fulfil the stroke definition above, the stroke will be classified as ischemic for purposes of the study.

# BLEEDING EVENTS

The overall safety objective of this study is to assess the safety and tolerability of long-term therapy with ticagrelor compared to placebo in patients with T2DM at increased risk of developing atherothrombotic events, with or without background low-dose ASA therapy.

Bleeding events will be analyzed using the Thrombolysis in Myocardial Infarction Study Group (TIMI), PLATO (PLATelet inhibition and patient Outcomes), and the Bleeding Academic Research Consortium (BARC) definitions. Specific focus will be on:

- Time to first TIMI major bleeding event following randomization (primary safety objective)
- Time to first TIMI Major or Minor bleeding event
- Time to first PLATO Major bleeding event
- Time to permanent discontinuation of study medication due to any bleeding event

## Bleeding Assessments

For all reported bleeding events relevant information will be compiled and sent to the CEC for central adjudication according to the following bleeding definitions: TIMI, PLATO (Wallentin L et al N Engl J Med 2009;361:1045-57) and BARC (Mehran R et al Circulation 2011;123:2736-47).

Additionally, all bleeding events (including hemorrhagic stroke) fulfilling the SAE criteria will be reported as SAEs by the sponsor to the authorities. If the adjudication of a potential endpoint event unveils a bleeding, e.g. hemorrhagic stroke not yet reported as SAE, it will be reported as an SAE by AZ to the authorities.

## Bleeding Associated with Procedures

Bleeding associated with procedures should only be reported as a bleeding event and AE/SAE if it exceeds what can be expected for the procedure.

**Calculation of change in hemoglobin or hematocrit in case of blood transfusion** (applies to all bleeding criteria).

To account for blood transfusions, hemoglobin (Hgb) and hematocrit (Hct) measurements will be adjusted for any packed red blood cells (PRBC) or whole blood given between baseline and post-transfusion measurements. A transfusion of one unit of blood will be assumed to result in an increase by 1 g/dL in hemoglobin; a transfusion of one unit of blood will be assumed to result in an increase of 3 percentage points in hematocrit. Thus, to calculate the true change in hemoglobin or hematocrit, if there has been an intervening transfusion between two blood measurements, the following calculations should be performed:

Δ Hgb = [Baseline Hgb – Post-transfusion Hgb] + [# of transfused units] Δ Hct = [Baseline Hct – Post-transfusion Hct] + [# of transfused units×3]

##

## TIMI Bleeding Criteria

1. **Major**

- Any intracranial bleeding (excluding microhemorrhages <10 mm evident only on gradient-echo MRI)
- Clinically overt signs of hemorrhage associated with a drop in hemoglobin of ≥5 g/dL or a ≥15% absolute decrease in hematocrit
- Fatal bleeding (bleeding that directly results in death within 7 d)
- If CABG related: fatal bleeding **or** perioperative intracranial bleeding **or** reoperation following closure of the sternotomy incision for the purpose of controlling bleeding **or** transfusion of ≥5 units of whole blood or PRBCs within a 48-hour period (cell saver transfusion will not be counted in calculations of blood products) **or** chest tube input >2 L within a 24-hour period.

1. **Minor**

- Clinically overt (including imaging), resulting in hemoglobin drop of 3 to <5 g/dL or ≥10% to <15% decrease in hematocrit
- No observed blood loss: ≥4 g/dL decrease in the hemoglobin concentration or

≥12% decrease in hematocrit

1. **Medical Attention**

Any overt sign of hemorrhage that meets one of the following criteria and does not meet the criteria for a major or minor bleeding event, as defined above:

- Requiring intervention: defined as medical practitioner-guided medical or surgical treatment to stop or treat bleeding including temporarily or permanently discontinuing or changing the dose of a medication or study drug
- Leading to hospitalization: defined as leading to or prolonging hospitalization
- Prompting evaluation: defined as leading to unscheduled contact with a healthcare professional and diagnostic testing (laboratory or imaging)

1. **Minimal**

- Any overt bleeding event that does not meet the criteria above
- Any clinically overt sign of hemorrhage (including imaging) associated with a

<3 g/dL decrease in hemoglobin concentration or <9% decrease in hematocrit

## PLATO Bleeding Classification

1. **Major/Life-threatening:**

- Fatal
- Intracranial
- Intrapericardial with cardiac tamponade
- Resulting in hypovolemic shock or severe hypotension that requires pressors or surgery
- Clinically overt or apparent bleeding associated with decrease in hemoglobin >5 g/dL
- Requiring transfusion of ≥4 U whole blood or PRBCs
- If CABG related: fatal bleeding or perioperative intracranial bleeding or reoperation following closure of the sternotomy incision for the purpose of controlling bleeding **or** transfusion of ≥5 units of whole blood or PRBCs within a 48-hour period (cell saver transfusion will not be counted in calculations of blood products) **or** chest tube input >2 L within a 24-hour period.

**Other Major**

- Significantly disabling (e.g., intraocular with permanent vision loss)
- Associated drop in hemoglobin of 3 to 5 g/dL
- Requiring transfusion of 2 to 3 U whole blood or PRBCs

1. **Minor**

Requiring medical intervention to stop or treat bleeding (e.g., epistaxis requiring visit to medical facility for packing)

1. **Minimal**

All others (e.g., bruising, bleeding gums, oozing from injection sites) not requiring intervention or treatment

## BARC Bleeding Definitions

**Type 0:** No evidence of bleeding

**Type 1*:** Bleeding that is not actionable and does not cause the patient to seek unscheduled performance of studies, hospitalization, or treatment by a health-care professional; may include episodes leading to self-discontinuation of medical therapy by the patient without consulting a health-care professional.

**Type 2*:** Any overt, actionable sign of hemorrhage (e.g., more bleeding than would be expected for a clinical circumstance, including bleeding found by imaging alone) that does not fit the criteria for type 3, 4, or 5 but does meet at least one of the following criteria:

- requiring nonsurgical, medical intervention by a health-care professional,
- leading to hospitalization or increased level of care, or
- prompting evaluation

*Predefined hierarchy for available data sources

Hierarchy for BARC type 1 and type 2 bleed:

- Source documentation, information in medical records overrides everything else
- BLEEDEV narrative or SAE description if serious AE
- DOSEDISC/DOSE – contains main reason for action taken with study drug, e.g. due to AE, subject decision
- Other data

If there are insufficient data available to decide between BARC type 1 or type 2, the event should be upgraded to BARC type 2 as default.

**Type 3:** Clinical, laboratory, and/or imaging evidence of bleeding with specific healthcare provider responses, as listed below:

## Type 3a:

- Overt bleeding plus hemoglobin drop of 3 to < 5 g/dL (provided hemoglobin drop is related to bleed)
- Any transfusion with overt bleeding

## Type 3b:

- Overt bleeding plus hemoglobin drop ≥5 g/dL (provided hemoglobin drop is related to bleed),
- Cardiac tamponade,
- Bleeding requiring surgical intervention for control (excluding dental/nasal/skin/ hemorrhoid),
- Bleeding requiring intravenous vasoactive agents

## Type 3c:

- Intracranial hemorrhage (does not include microbleeds or hemorrhagic transformation, does include intraspinal),
- Subcategories confirmed by autopsy or imaging or lumbar puncture,
- Intraocular bleed compromising vision.

**Type 4:** CABG-related bleeding

- Perioperative intracranial bleeding within 48 h,
- Reoperation after closure of sternotomy for the purpose of controlling bleeding
- Transfusion of ≥ 5 U whole blood or packed red blood cells within a 48-h period,
- Chest tube output more than or equal to 2L within a 24-h period

**Type 5:** Fatal bleeding

- **Type 5a**: Probable fatal bleeding; no autopsy or imaging confirmation but clinically suspicious
- **Type 5b:** Definite fatal bleeding; overt bleeding or autopsy or imaging confirmation
